# *Bacillus subtilis* spores as adjuvants against avian influenza H9N2 induce antigen-specific antibody and T cell responses in White Leghorn chickens

**DOI:** 10.1186/s13567-020-00788-8

**Published:** 2020-05-24

**Authors:** Ji Eun Lee, Yoon-Chul Kye, Sung-Moo Park, Byoung-Shik Shim, Sungsik Yoo, Eunmi Hwang, Hyungkuen Kim, Sung-Jo Kim, Seung Hyun Han, Tae Sub Park, Byung-Chul Park, Cheol-Heui Yun

**Affiliations:** 1 Department of Agricultural Biotechnology and Research Institute of Agriculture and Life Sciences, Seoul National University, Seoul, Republic of Korea; 2 Center for Food and Bioconvergence, Seoul National University, Seoul, Republic of Korea; 3 G+FLAS Life Sciences, Seoul, Republic of Korea; 4 Choong-Ang Vaccine Laboratory, Daejeon, Republic of Korea; 5 Department of Biotechnology, Hoseo University, Asan, Chungcheongnam-do Republic of Korea; 6 Department of Oral Microbiology and Immunology, DRI and BK21 Program, School of Dentistry, Seoul National University, Seoul, Republic of Korea; 7 Graduate School of International Agricultural Technology, Institute of Green-Bio Science and Technology, Seoul National University, Pyeongchang-gun, Gangwon-do Republic of Korea

## Abstract

Low-pathogenicity avian influenza H9N2 remains an endemic disease worldwide despite continuous vaccination, indicating the need for an improved vaccine strategy. *Bacillus subtilis* (*B. subtilis*), a gram-positive and endospore-forming bacterium, is a non-pathogenic species that has been used in probiotic formulations for both animals and humans. The objective of the present study was to elucidate the effect of *B. subtilis* spores as adjuvants in chickens administered inactivated avian influenza virus H9N2. Herein, the adjuvanticity of *B. subtilis* spores in chickens was demonstrated by enhancement of H9N2 virus-specific IgG responses. *B. subtilis* spores enhanced the proportion of B cells and the innate cell population in splenocytes from chickens administered both inactivated H9N2 and *B. subtilis* spores (Spore + H9N2). Furthermore, the H9N2 and spore administration induced significantly increased expression of the pro-inflammatory cytokines IL-1β and IL-6 compared to that in the H9N2 only group. Additionally, total splenocytes from chickens immunized with inactivated H9N2 in the presence or absence of *B. subtilis* spores were re-stimulated with inactivated H9N2. The subsequent results showed that the extent of antigen-specific CD4^+^ and CD8^+^ T cell proliferation was higher in the Spore + H9N2 group than in the group administered only H9N2. Taken together, these data demonstrate that *B. subtilis* spores, as adjuvants, enhance not only H9N2 virus-specific IgG but also CD4^+^ and CD8^+^ T cell responses, with an increase in pro-inflammatory cytokine production. This approach to vaccination with inactivated H9N2 together with a *B. subtilis* spore adjuvant in chickens produces a significant effect on antigen-specific antibody and T cell responses against avian influenza virus.

## Introduction

Avian influenza has been a global problem not only because it infects wild and domestic birds but also because it can be transmitted to humans. One of the low-pathogenicity avian influenza viruses, H9N2, does not induce severe pathology in birds or humans compared to that induced by highly pathogenic viruses; however, it has been focused on for decades because of its economic damage in the poultry industry. Since it was first identified in 1966 [1], H9N2 has become endemic worldwide, especially in Asia and Africa. Some countries, including China, Republic of Korea, and Egypt, have adopted a vaccination scheme against H9N2 in their poultry farms [2]. However, H9N2 outbreaks have been continuously reported even in farm animals immunized against avian influenza [3], implying that the current vaccination strategy is in need of advancement for improved performance. This could be due to the antigenic shift and drift of viruses, weak antigenicity of current vaccines and/or inappropriate vaccination strategy in poultry farms [4].

With growing interest in the importance of the gut microbiota, probiotics that contain beneficial bacteria or yeast have also been tried in the domestic animal industry. A large number of field studies have shown the positive effect of probiotics on growth performance or the immune system [5, 6]. In particular, *Bacillus subtilis*, a gram-positive bacterium, is a non-pathogenic species that has also been used as a probiotic for both animals and humans as feed [7] or health food [8], respectively. It is indeed classified as a generally regarded as safe microorganism by the Food and Drug Administration. *B. subtilis* is an endospore-forming bacterium that can differentiate into a form of dormant spores under harsh environmental conditions, including nutrient starvation and extreme thermal changes [9]. Sporulation initiates when DNA segregation is completed and concurrently with the asymmetric invagination of the membrane by forming a polar septum near one pole of the cell [10, 11]. Then, the immature spore stage (i.e., the forespore) is surrounded by a double membrane of the mother cell and develops into the mature spore [10].

In previous studies, *B. subtilis* spores showed potential for use as an adjuvant in mice. *B. subtilis* spores not only enhance innate immunity that protects against respiratory infections [12–14] but also induce an increase in antigen-specific antibody and T cell responses when co-administered with a soluble antigen [15–17]. *B. subtilis* spore-induced cross-presentation in response to a co-administered antigen suggests that the spore instructs diverse antigen-specific adaptive immune responses [15, 18]. Other reports also suggested that genetically modified *B. subtilis* spores displaying antigens on their surface can enhance antibacterial or antiviral immunity [16, 19–23]. An additional advantage of *B. subtilis* spores as adjuvants in influenza vaccines includes the enhanced effect of the vaccine and the reduced frequency of immunization required for the optimal immune response for full protection [24, 25]. A previous study demonstrated that *B. subtilis* spores could be a viable vaccine adjuvant against influenza in mice [13], with a reservation for safety and efficacy issues for further empirical investigation. Thus, we explored the ability of *B. subtilis* spores to influence the diversity of immune responses induced by inactivated H9N2 avian influenza virus in chickens. Specifically, we attempted to elucidate the mechanism for intrinsic induction of humoral and cell-mediated immune responses in chickens immunized with inactivated H9N2 and *B. subtilis* spores as adjuvants.

*B. subtilis* spores have been suggested as probiotics against enteric pathogens in chickens [7, 14]. However, it is important to note that very few studies using *B. subtilis* spores as vaccine adjuvants have been performed in the poultry field. Therefore, in the present study, we examined whether the *B. subtilis* spores work as adjuvants against influenza based on the induction of B cell and T cell responses in chickens.

## Materials and methods

### Chickens

Fertile eggs from White Leghorn chickens were provided by University Animal Farm, College of Agriculture and Life Sciences, Seoul National University (Pyeongchang, Republic of Korea). The eggs were incubated in a 37.5–38 °C incubator (Rcom, Gimhae, Republic of Korea) for 21 days. Five chickens were allotted to each group. The care room was maintained at 23–25 °C, with 40% humidity under positive pressure. Hatched chickens were raised under conventional conditions and were allowed free access to feed and water. The experiment was approved by the Institutional Animal Care and Use Committee of Seoul National University (SNU-150327-2-1).

### Preparation and isolation of *Bacillus subtilis* spores

*Bacillus subtilis* strain HB3 (National Culture Collection for Pathogen, Republic of Korea) was spread on an agar plate containing 3% trypticase soy broth (TSB), 0.5% yeast extract (YE) and 1.5% Bacto agar (all from BD Biosciences, San Jose, USA) and incubated at 37 °C for 9 h. One colony was randomly picked and inoculated in 25 mL of 3% TSB and 0.5% YE liquid medium. Then, the culture was incubated for 5 h in a shaking incubator (BioFree, Seoul, Republic of Korea) at 37 °C until the OD value reached between 0.45 and 0.6. For sporulation, the culture was transferred to 500 mL of autoclaved 3% TSB and 0.5% YE medium containing 5 mL of 10% KCl, 5 mL of 1.2% MgSO_4_.7(H_2_O) (pH 7.6), and 0.5 mL of 1 M Ca(NO_2_)_3_, 0.01 M MnCl_2_, and 1 mM FeSO_4_. The culture was incubated at 37 °C for 48 h in a shaking incubator. The cells were collected by centrifugation at 5516 × *g* for 10 min, re-suspended in distilled water, and incubated at 4 °C for 48 h on a rocker. Then, the cells were sonicated at 35% amplitude (1 Watt) for 90 s with a 0.5 s pulse. Spores, loaded on an OptiPrep density gradient (Sigma-Aldrich, St. Louis, USA) with layers of 35%, 25%, and 15%, were centrifuged at 10 000 × *g* for 40 min at 25 °C without disruption for purification. The *B. subtilis* spores were washed three times and re-suspended in PBS.

### H9N2 virus inactivation

H9N2 influenza virus (A/Chicken/Korea/01310/2001, strain CE20, from Prof. Jae Hong Kim, College of Veterinary Medicine, Seoul National University) was inactivated with formalin at 37 °C for 18 h at a final concentration of 0.1% [26]. The formalin was neutralized by the addition of NaHSO_3_ solution to the inactivated virus as previously described [13].

### Immunization schedule

One-week-old White Leghorn chickens were immunized with phosphate-buffered saline (PBS), *B. subtilis* spores, inactivated H9N2, or both *B. subtilis* spores and inactivated H9N2 in a volume of 200 μL. For vaccination, 1 × 10^8^ EID_50_ of H9N2 viruses/200 μL per animal was administered intramuscularly. For comparison with a commercial vaccine, the same dose and strain of commercial H9N2 inactivated oil vaccine (KBNP, Anyang, Republic of Korea) was used in the oil-vaccine group. Randomly selected White Leghorn chickens were allotted into five different groups (four to five animals per group) as follows: PBS control (Con) as a negative control, 2 × 10^9^ CFU of *B. subtilis* spores alone (Spore), inactivated H9N2 alone (H9N2), inactivated H9N2 together with *B. subtilis* spores (Spore + H9N2) or commercial H9N2 oil vaccine (Oil vaccine). The immunization regimen comprised two injections via the intramuscular (i.m.) route at 7 and 14 days of age. Blood samples were collected at 7 and 14 days (i.e., 21 and 28 days old) after the last immunization from the wing vein, and serum was collected with centrifugation at 10 000 × *g* for 10 min at 4 °C to analyse the antigen-specific antibody responses. Spleens were collected and used for in vitro culture and flow cytometric analysis, as explained below.

### Serum antibody detection

Antigen-specific IgG in serum was analysed by ELISA. For H9N2-specific IgG, 100 μL of formalin-inactivated H9N2 influenza A virus per well was used for coating onto a 96-well microplate (Thermo, Waltham, USA) overnight at 4 °C. Serially diluted (5-fold) sera along with controls were incubated for 2 h at room temperature, followed by a 1 h incubation with 100 μL of rabbit anti-chicken IgG conjugated with HRP at a 1:50 000 dilution (Bethyl Laboratories, Montgomery, USA). After incubation for 1 h at room temperature, TMB (Merck, Darmstadt, Germany) was added until colour developed, and then the reaction was stopped by the addition of 50 μL of 2 N H_2_SO_4_. Absorbance was measured at 450 nm using an ELISA microplate reader (Molecular Devices, San Jose, USA).

### Haemagglutination inhibition assay

The haemagglutination inhibition (HI) titre was determined by using chicken erythrocytes collected with Alsever’s solution (Sigma-Aldrich). Serially 2-fold diluted sera (25 μL/well) from each group of chickens (four to five animals per group) at 4 weeks old were incubated with 25 μL of H9N2 virus (4 HAU)/well in a U-bottom 96-well plate (Thermo) for 30 min at room temperature. Chicken erythrocytes (50 μL/well) were added and incubated for 30 min at room temperature. Then, the plate was analysed to distinguish agglutinated from non-agglutinated wells, of which the highest dilution showing clear red dots was determined as the HI titre.

### In vitro T cell receptor (TCR) stimulation

Splenocytes from 3-week-old chickens were stained with a mouse anti-chicken CD3 antibody (Southern Biotechnology, Birmingham, USA) followed by incubation with anti-mouse IgG beads (Miltenyi Biotec, Bergisch Gladbach, Germany) for 20 min. CD3^+^ T cells were isolated by MACS magnetic bead sorting (Miltenyi Biotec) and stained with 1 μM CellTrace™ Violet (CTV) dye (Invitrogen, Waltham, USA) for 25 min at 37 °C. Then, the cells were washed with pre-warmed complete medium. CD3^+^ T cells stained with CTV were cultured in anti-chicken CD3 and CD28 antibody-coated 96 flat-bottom plates (Thermo) for 2 days. For some experiments, chicken monocyte/macrophage cells were isolated by using mouse anti-chicken monocyte/macrophage (KUL01) antibodies followed by anti-mouse microbead (Miltenyi Biotec) and MACS separators. Then, the cells were stimulated with inactivated H9N2 and/or *B. subtilis* spores, and the supernatants were collected after centrifugation at 300 × *g* for 10 min. The supernatant was treated with T cells together with anti-CD3/CD28 antibodies. The cells were stained with anti-chicken CD4 and CD8 antibodies, and proliferative activity was determined by flow cytometry (FACS Canto II, BD Biosciences) and analysed using FlowJo software (Tree star, Ashland, USA). Division index scores were calculated manually according to the following equation suggested by FlowJo software (Tree Star), as adopted from the previous report [27]:

$${\text{Division index}} = \frac{{\mathop \sum \nolimits_{0}^{i} i \times \frac{{N_{i} }}{{2^{i} }}}}{{\mathop \sum \nolimits_{0}^{i} \frac{{N_{i} }}{{2^{i} }}}}$$

where *i* is the generation number and *N*_*i*_ is the number of cells in generation *i*.

### Single cell dissociation

The spleen was collected, minced and filtered through a 70-μm nylon cell strainer (Corning, New York, USA) to obtain a single cell suspension. The splenocytes were then suspended in 5 mL of RPMI 1640 containing heat-inactivated 5% (vol/vol) FBS and 1% (vol/vol) antibiotics/antimycotic solution (all from Invitrogen) and centrifuged at 300 × *g* for 3 min at 4 °C. Then, the pellet was treated with 1 mL of ACK lysing buffer (Thermo) incubated for 3 min at room temperature and centrifuged at 300 × *g* for 3 min at 4 °C. The pellet was washed and re-suspended in medium and filtered through a 70-μm strainer.

### Flow cytometric analysis

A single-cell suspension of total splenocytes was stained for 20 min at 4 °C in the dark with a combination of the following fluorochrome-conjugated monoclonal antibodies: anti-chicken CD3-PACBLU (CT-3), anti-CD4-FITC (CT-4), anti-CD8α-PE (CT-8), anti-Bu-1-Alexa Fluor^®^ 647 (AV20), and anti-monocyte/macrophage-PE (KUL01) (Southern Biotechnology). An anti-7AAD-PerCP-Cy5.5 antibody was purchased from BD Biosciences. After staining, the cells were washed, and the expression of surface markers was measured by flow cytometry (FACS Canto II). All the flow cytometric data were analysed using FlowJo software (Tree star).

### Re-stimulation with H9N2 and purification of CD3^+^ T cells by magnetic beads

To analyse the antigen-specific T cells in the spleen, splenocytes were collected from vaccinated chickens 7 days after the last immunization. Splenocytes were re-stimulated with 32 HAU of inactivated H9N2 [26] for 24 h. Then, splenocytes were stained with an anti-chicken CD3 (CT3) antibody (Southern Biotechnology). After washing with MACS buffer (PBS containing 0.5% BSA and 2 mM EDTA), the cells were incubated with anti-mouse IgG microbeads (Miltenyi Biotec) for 15 min in the dark and centrifuged at 300 × *g* for 10 min at 4 °C. Then, the cell suspension was separated on a MACS LS column that was placed in the magnetic field of a MACS Separator (Miltenyi Biotec). The magnetic fraction of positively selected CD3^+^ cells was used in the mRNA experiments for IFN-γ, IL-17 and IL-4.

### RNA extraction and cDNA synthesis

Total RNA was extracted from splenocytes or purified T cells using NucleoZOL (Machery-Nagel, Duren, Germany) according to the manufacturer’s instructions. Briefly, single cells of the splenocytes or T cells were treated with 1 mL of NucleoZOL per 5 × 10^6^ cells. Total RNA was isolated by the addition of 400 μL of RNase-free water (Sigma-Aldrich) followed by centrifugation at 12 000 × *g* for 15 min. Then, 500 μL of aqueous phase was transferred into a new tube, and the same volume of isopropanol was added. Next, the samples were incubated for 10 min at room temperature for RNA precipitation and centrifuged at 12 000 × *g* for 10 min. The RNA pellet was obtained at the bottom of the tube after washing with 75% ethanol followed by air drying for 5–10 min and resuspension with RNase-free water. RNA concentration was quantified with a NanoDrop (Amersham Biosciences, Buckinghamshire, UK) at A260. Subsequently, 500 ng of purified RNA was reverse transcribed to cDNA using M-MLV reverse transcriptase (Invitrogen) according to the manufacturer’s instructions.

### Real-time quantitative PCR

Real-time quantitative PCR was performed on cDNA using a StepOne Plus real-time PCR system (Applied Biosystems, Waltham, USA). SYBR^®^ Green PCR Master Mix was used according to the manufacturer’s specification (Applied Biosystems). PCR was carried out in a 96-well reaction plate with 10 μL of SYBR^®^ Green PCR master mix, 0.5 μL of primers, 1–2 μL of cDNA template and 7–8 μL of nuclease-free H_2_O. Each reaction involved a pre-incubation at 95 °C for 10 min, followed by 40 thermal cycles at 95 °C for 15 s, 55 °C for 30 s and elongation at 72 °C for 30 s. Relative quantification of target genes was calculated using the 2^−ΔΔCt^ method. Target gene expression was normalized to the β-actin mRNA level. Primer sequences used for real-time quantitative PCR (Table 1) were designed using NCBI Primer-BLAST and synthesized by Bioneer Inc. (Daejeon, Republic of Korea).

**Table 1:** **Primer sequences used for real-time quantitative PCR**

Target gene	Primer sequence	Product size (bp)
β-actin	F: CAACACAGTGCTGTCTGGTGGTA R: ATCGTACTCCTGCTTGCTGATCC	205
BAFF	F: CACGTCATCCAGCAGAAGGAT R: ACAAGAGGACAGGAGCATTGC	120
BAFF-R	F: CCTGGCCCCACCATAAGG R: CATTACAGTCTCTCCTCACCCATACA	120
CD40	F: TGCACACCCTGTGAGAATGGT R: CGTTGCGTTTCCCTGTCTCTT	120
CD40L	F: TGAAGTGGATGACGACGAGCTA R: TGGTGCAGAAGCTGACTTGTG	120
TACI	F: GGCTCCTCATCCCAGTTCCT R: TTGTGCGTGAAGAAAGCTCTGT	120
IL-1β	F: GCTCTACATGTCGTGTGTGATGAG R: TGTCGATGTCCCGCATGA	80
IL-4	F: AACATGCGTCAGCTCCTGAAT R: TCTGCTAGGAACTTCTCCATTGAA	98
IL-6	F: GCTCGCCGGCTTCGA R: GGTAGGTCTGAAAGGCGAACAG	71
IL-15	F: TAGGAAGCATGATGTACGGAACAT R: TTTTTGCTGTTGTGGAATTCAACT	83
IL-17	F: GCTGCAGCAAGAAAAGGAAGA R: GCCGTATCACCTTCCCATGT	120
IFN-γ	F: AACCTTCCTGATGGCGTGAA R: GCTTTGCGCTGGATTCTCAA	86

### Statistical analysis

All data are expressed as the mean values ± standard deviations (SDs). For comparison of means between two groups, the data were analysed using two-tailed, paired Student’s *t* test and considered statistically significant when the *P*-value was less than 0.05. For multiple group comparisons, one-way ANOVA followed by a Friedman test was applied. All statistical analyses were performed using GraphPad Prism (version 5.01, GraphPad Software, Inc., San Diego, USA).

## Results

### Enhancement of H9N2 virus-specific IgG production in chickens immunized with inactivated H9N2 with *B. subtilis* spores

**Figure 1:**
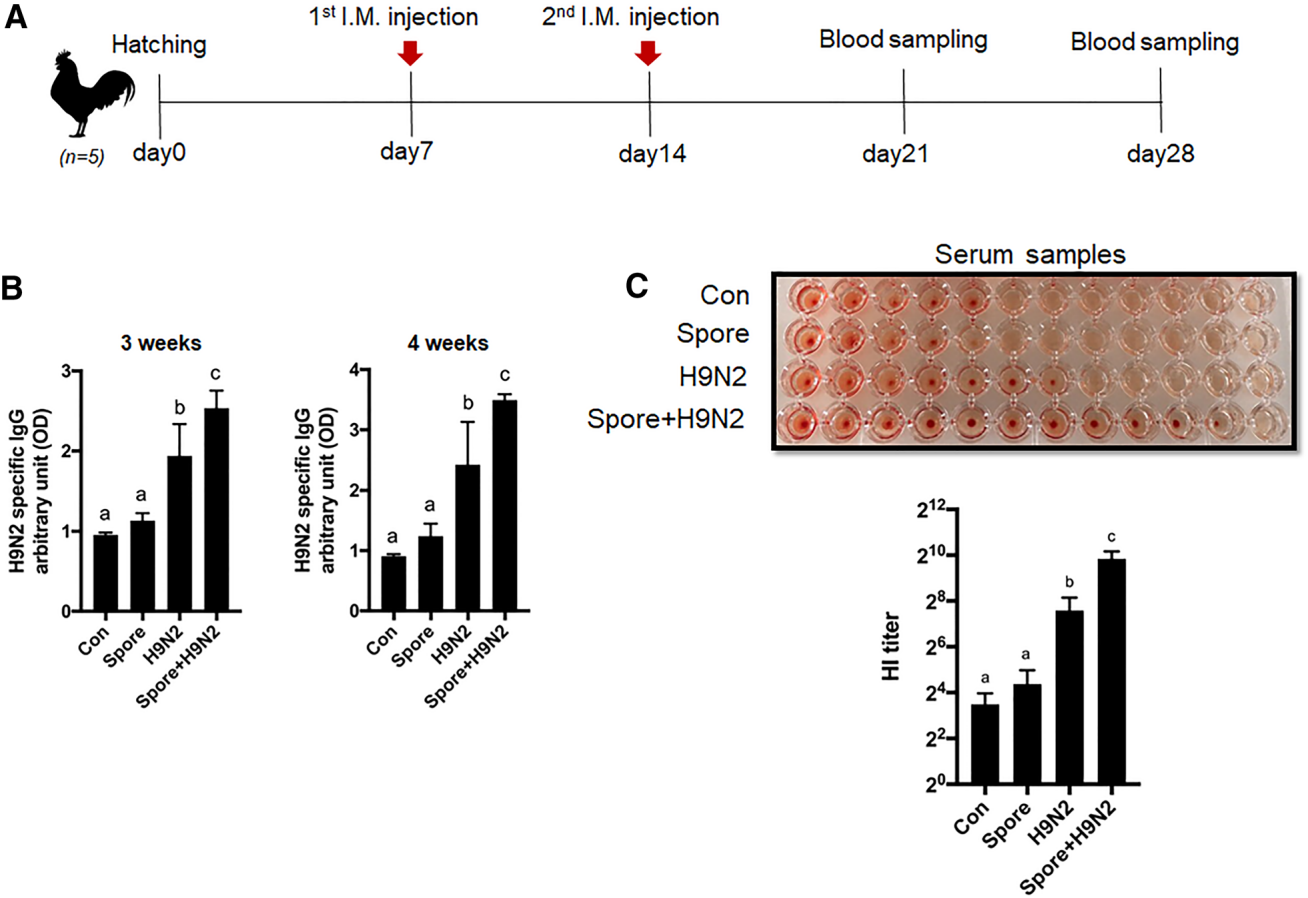
**Antigen-specific IgG response and HI titre in chickens with intramuscular administration of inactivated H9N2 and/or*B. subtilis*spores. A** Scheme of the immunization schedule. One-week-old White Leghorn chickens (*N* = 5) were administered with PBS as a negative control, 2 × 10^9^ CFU of *B. subtilis* spores, or inactivated H9N2 without or with *B. subtilis* spores. **B** The antigen-specific IgG antibody response in serum was measured and expressed as arbitrary units at 3 and 4 weeks. **C** HI assays with sera against H9N2 virus at 4 weeks. To determine the significance, one-way ANOVA followed by a Friedman test corrected by Dunn’s multiple comparison test was performed. Data are expressed as the mean values ± SDs. Different letters on each group denote a significant difference at *P* ≤ 0.05.

To determine the adjuvant effect of *B. subtilis* spores on antigen-specific antibody responses, chickens were intramuscularly immunized with inactivated H9N2 with or without *B. subtilis* spores according to the immunization schedule (Figure 1A). None of the chickens at 28 days after hatching showed abnormalities, signs of illness, body weight loss or death during the experimental period (data not shown). Serum H9N2 virus-specific IgG production in chickens immunized with inactivated H9N2 and *B. subtilis* spores (Spore + H9N2) was significantly higher than that of other groups at both day 21 and day 28 (Figure 1B). To investigate the virus-specific inhibition of serum antibodies, we conducted a HI assay against H9N2 virus with serum from day 28. The results showed that the HI titre of Spore + H9N2 group was significantly higher than those of the other groups (Figure 1C). Collectively, these results suggested that *B. subtilis* spores act as adjuvants in chickens, enhancing antigen-specific immune responses when immunized with the inactivated H9N2 avian influenza virus.

### Changes in B cells and monocyte/macrophage subsets in chickens administered Spore + H9N2

**Figure 2:**
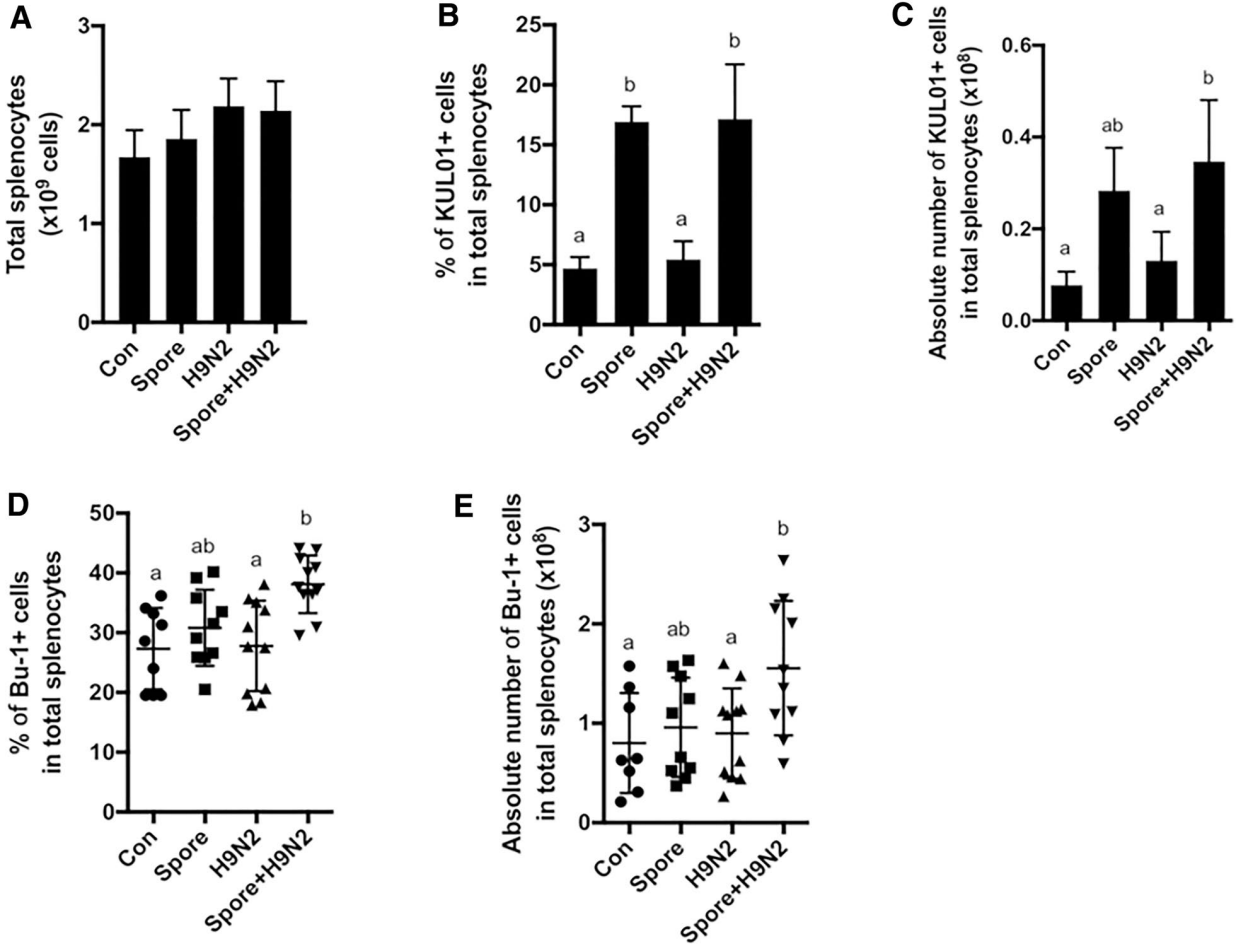
**Analysis of splenic B cells and innate immune cells in chickens administered inactivated H9N2 and*B. subtilis*spores.** Seven-day-old chickens (*N* = 10) were immunized twice with inactivated H9N2 and/or *B. subtilis* spores at one-week intervals. Single cells from splenocytes were stained with the proper combination of anti-chicken CD3, Bu-1, and KUL01 antibodies, and flow cytometric analysis was performed. **A** Total splenocytes, **B** the percentage and **C** absolute number of KUL01^+^ monocytes/macrophages cells, and **D** the percentage and **E** absolute number of Bu-1^+^ cells were examined by using flow cytometry. To determine the significance, one-way ANOVA followed by a Friedman test corrected by Dunn’s multiple comparison test was performed. Data are expressed as the mean values ± SDs. Different letters on each group denote a significant difference at *P* ≤ 0.05.

We next investigated the changes in immune cells in the spleens of chickens immunized with inactivated H9N2 and/or *B. subtilis* spores. The total cell number of splenocytes seemed to increase slightly, but there were no significant changes among the groups (Figure 2A). However, administration of *B. subtilis* spores led to an increase in the percentage (Figure 2B) and absolute number (Figure 2C) of KUL01^+^ monocyte/macrophage populations compared to those of these populations in the H9N2 group.

We next analysed the changes in B cells, which are major adaptive immune effectors producing antigen-specific antibodies. The percentage (Figure 2D) and absolute number (Figure 2E) of the Bu-1^+^ B cell population were significantly increased only in the Spore + H9N2 group but not in the H9N2 group or Spore group after the second immunization. These results suggested that *B. subtilis* works as an adjuvant to enhance the proliferation of B cells when co-administered with the H9N2 antigen. Collectively, *B. subtilis* spores efficiently activated innate immune cells by themself and increased the proportion and number of B cells when administered with antigens.

### Gene expression patterns in splenocytes treated with *B. subtilis* spores

**Figure 3:**
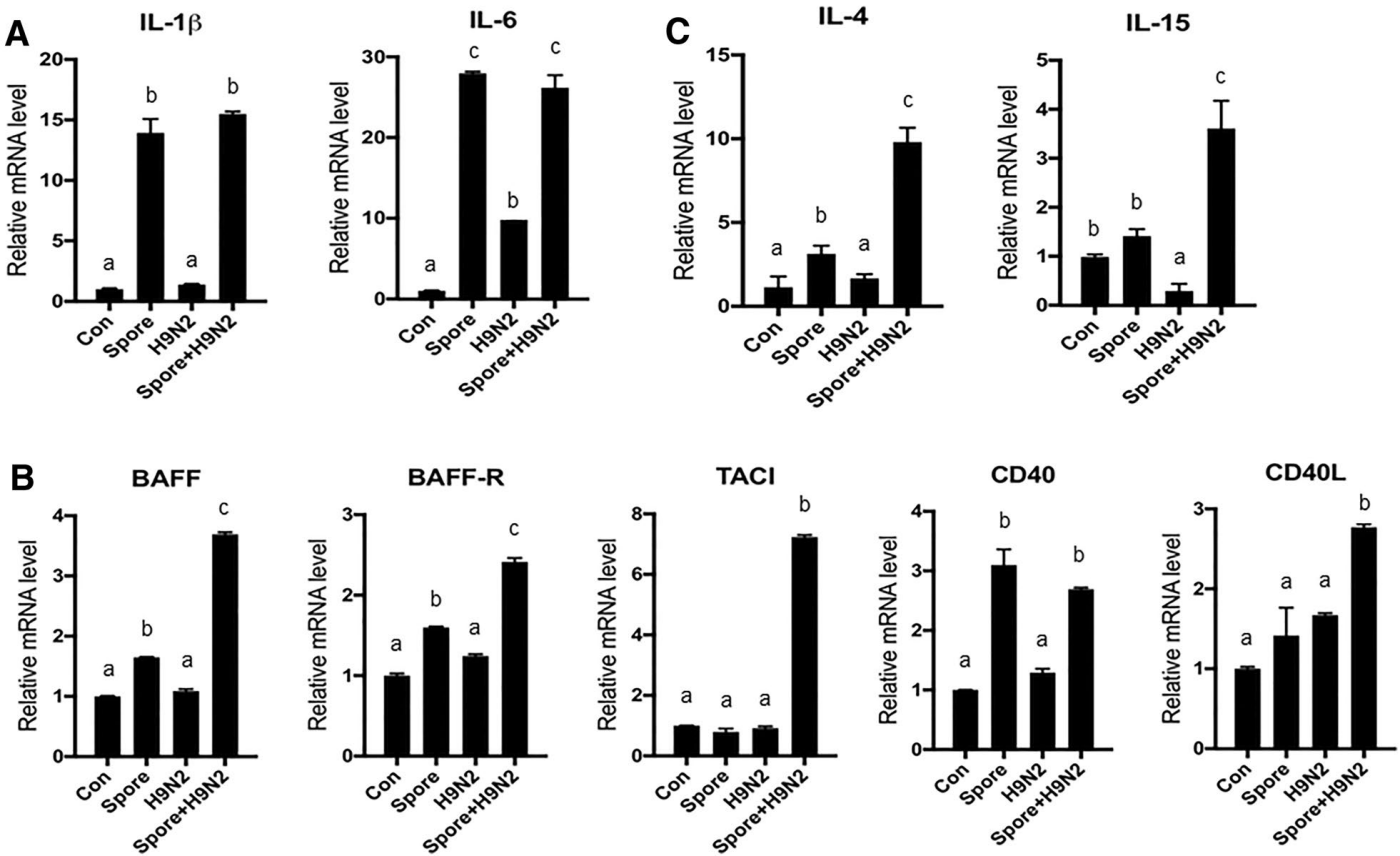
**mRNA expression patterns of pro-inflammatory cytokines in monocytes/macrophages and B cell proliferation/survival-related genes in chicken splenocytes treated with inactivated H9N2 and*B. subtilis*spores**. Total splenocytes and monocytes/macrophages were isolated from 3-week-old chickens (*N* = 5) and stimulated with inactivated H9N2 and/or *B. subtilis* spores for 3 h. **A** The expression patterns of the IL-1 and IL-6 genes, as pro-inflammatory cytokines, in monocytes/macrophages. **B** The mRNA expression levels of the B cell proliferation-related genes BAFF, BAFF-R, TACI, CD40, and CD40L in total splenocytes. **C** The expression patterns of the IL-4 and IL-15 genes in total splenocytes by qRT-PCR. To determine the significance, one-way ANOVA followed by a Friedman test corrected by Dunn’s multiple comparison test was performed. Data are expressed as the mean values ± SDs. Different letters on each group denote a significant difference at *P* ≤ 0.05.

Next, we sought to determine what kind of immune-related genes were upregulated in response to *B. subtilis* spores. To examine the gene expression pattern for pro-inflammatory cytokines, quantitative RT-PCR analysis was conducted in splenocytes treated with H9N2 and/or *B. subtilis* spores in vitro. The expression of IL-1β and IL-6, major pro-inflammatory cytokines produced in innate immune cells, was significantly increased in splenocytes stimulated with the *B. subtilis* spore adjuvant (Figure 3A). The mRNA expression levels of BAFF, BAFF receptor (BAFF-R), transmembrane activator and calcium-modulating cytophilin ligand interactor (TACI), CD40 and CD40L, which are responsible for the proliferation and survival signals in B cells, were higher in the Spore + H9N2 group than in the other groups (Figure 3B). In addition, the mRNA expression of IL-4 and IL-15, which are pro-survival factors for B cells or T cells, was significantly upregulated in the Spore + H9N2 group (Figure 3C). These results indicated that the expression of pro-inflammatory cytokines and key regulators of B cells was enhanced in splenocytes treated with the *B. subtilis* spore adjuvant.

### *B. subtilis* spore adjuvant promoted CD4^+^ and CD8^+^ T cell proliferation

**Figure 4:**
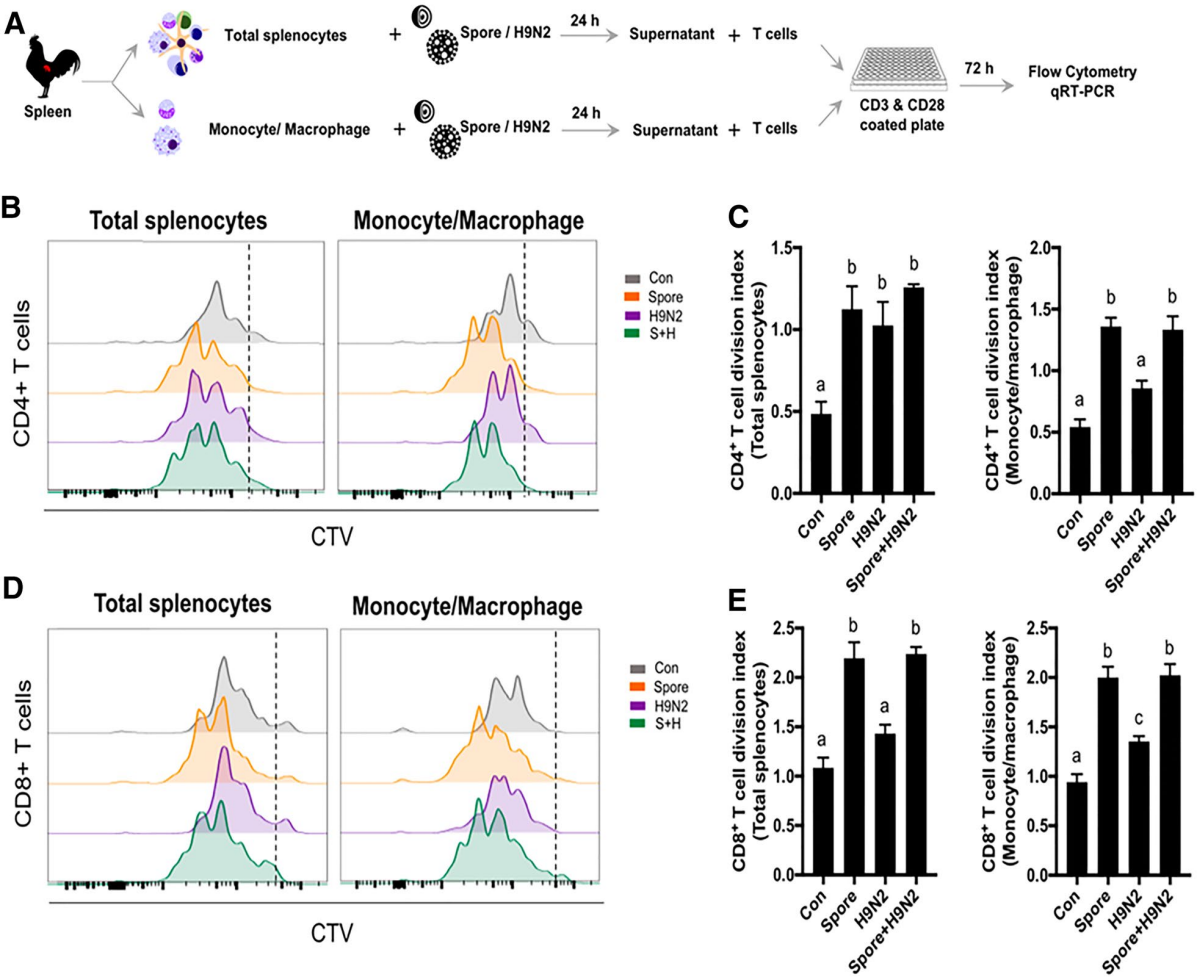
**CD4**^**+**^**and CD8**^**+**^**T cell proliferation after TCR stimulation co-treatment with the supernatant from splenocytes or monocytes/macrophages stimulated with inactivated H9N2 and*B. subtilis*spores**. **A** CTV-labelled purified CD3^+^ T cells were cultured in anti-CD3 and anti-CD28 antibody-coated plates with supernatants from splenocytes or monocytes/macrophages stimulated with inactivated H9N2 and/or *B. subtilis* and analysed by using flow cytometry. **B**–**E** The proliferative populations of CD4^+^ T cells (**B** and **C**) and CD8^+^ T cells (**D** and **E**) were measured by CTV histograms and division index scores. To determine the significance, one-way ANOVA followed by a Friedman test corrected by Dunn’s multiple comparison test was performed. Data are expressed as the mean values ± SDs. Different letters on each group denote a significant difference at *P* ≤ 0.05.

For the best T cell immunity, the combination of three components is necessary: TCR stimulation, co-stimulatory signalling and cytokines. Among them, we first examined the role of cytokines induced by *B. subtilis* spores. To test the role of cytokines from innate immune cells, we collected the supernatant from splenocytes or monocytes/macrophages stimulated with *B. subtilis* spores in vitro. CD3^+^ T cells from unimmunized chickens and labelled with CTV were treated with the supernatant and examined for proliferation [28]. TCR and co-stimulatory signalling was induced by using anti-CD3 and anti-CD28 antibodies together with the supernatant, as shown in the experimental scheme (Figure 4A). Flow cytometric analyses showed a strong CD4^+^ T cell proliferation when T cells treated with the supernatant from the *B. subtilis* spore-treated group (Figures 4B and C). Similar to total splenocytes, the supernatant from KUL01^+^ monocytes/macrophages stimulated with spores promoted CD4^+^ T cell proliferation (Figures 4B and C). In addition, CD8^+^ T cells cultured in the supernatant from *B. subtilis* spore-treated total splenocytes or monocytes/macrophages also showed high proliferative capacity regardless of the presence of H9N2 (Figures 4D and E). These results demonstrated that cytokines from innate cells induced by *B. subtilis* spores could further enhance the proliferation of CD4^+^ and CD8^+^ T cells.

### Changes in major immune cell populations induced by the spore-adjuvanted vaccine compared to those induced by a commercial oil adjuvant vaccine

**Figure 5:**
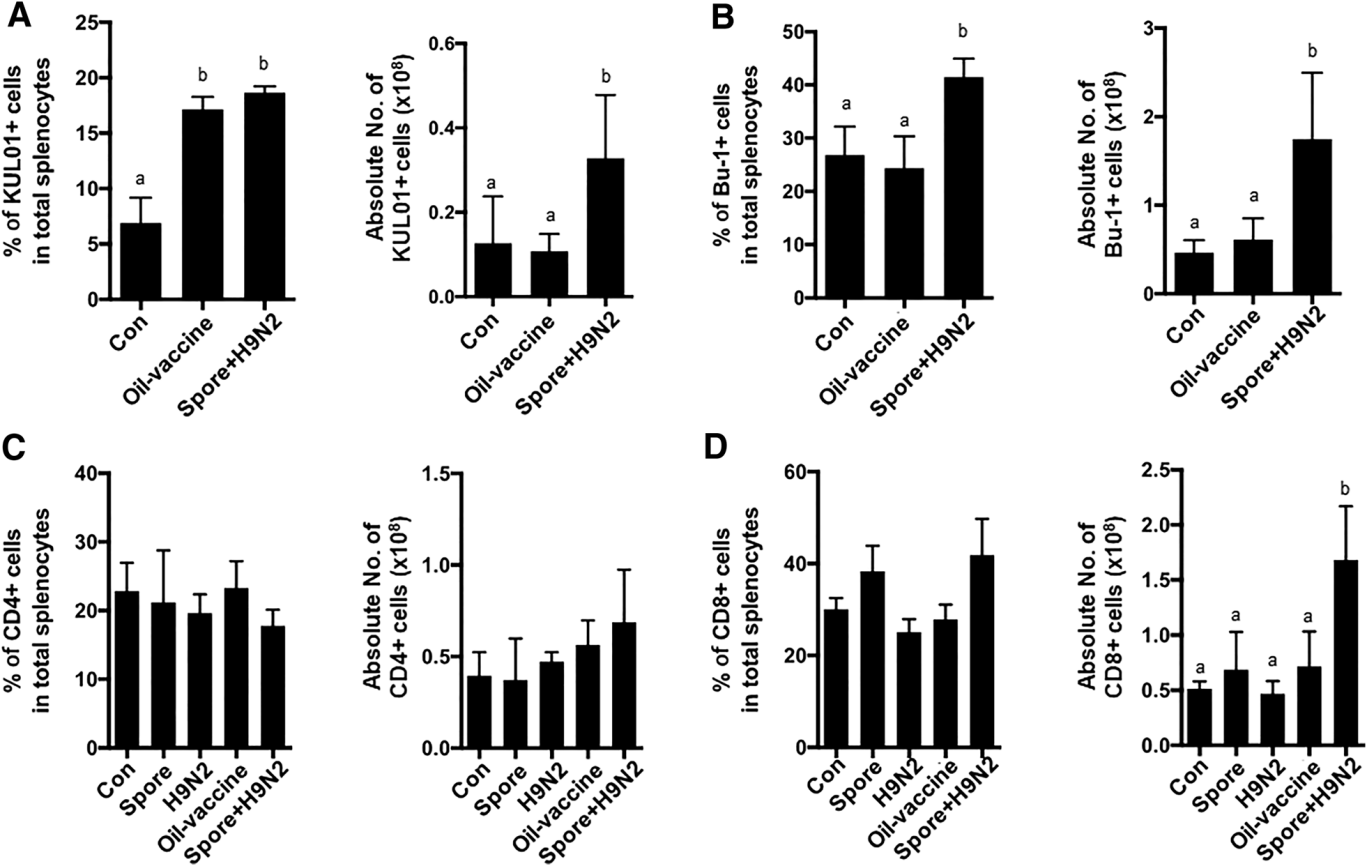
**Comparison of the immune cell proportion after administration of H9N2 with*B. subtilis*spores or a commercial vaccine.** Seven-day-old chickens (*N* = 5) were immunized twice with inactivated H9N2 and/or *B. subtilis* spores at one-week intervals. At one week post second immunization, single cells from splenocytes were stained with anti-KUL01, anti-Bu-1, anti-CD4 and anti-CD8 antibodies, and flow cytometry analysis was performed. The percentages and absolute numbers of **A** KUL01^+^ monocytes/macrophage cells, **B** Bu-1^+^ B cells, and **C** CD4^+^ and **D** CD8^+^ T cells were examined. To determine the significance, one-way ANOVA followed by a Friedman test corrected by Dunn’s multiple comparison test was performed. Data are expressed as the mean values ± SDs. Different letters on each group denote a significant difference at *P* ≤ 0.05.

Generally, oil adjuvant vaccines are used against H9N2 in poultry farms, but their limitations still exist. Since the treatment of *B. subtilis* spores together with inactivated H9N2 showed an increase in KUL01^+^ monocytes/macrophages (Figure 2B) and B cells (Figure 2D) in chickens, we compared the major immune cell changes with those of commercial H9N2 oil vaccine-immunized chickens. Chickens immunized with the commercial H9N2 oil vaccine or Spore + H9N2 chickens showed a significantly increased percentage of the KUL01^+^ monocyte/macrophage population compared to that in the control chickens (Figure 5A), yet the absolute number was significantly higher only in chickens immunized with Spore + H9N2 treatment (Figure 5A).

Both the proportion and the absolute number of the B cell populations were significantly higher in the Spore + H9N2 group than in the oil vaccine groups (Figure 5B). There were no significant differences in CD4^+^ T cells among the treated groups (Figure 5C), while the percentage and absolute number of the CD8^+^ T cell population were significantly higher in chickens immunized with Spore + H9N2 than in the oil vaccine group chickens (Figure 5D). These findings suggested that chickens administered the Spore + H9N2 vaccine showed a higher antigen-specific immune cell population than the chickens administered the commercial oil vaccine.

### *B. subtilis* spores as adjuvants promoted Th1- and Th17-derived cytokine expression

**Figure 6:**
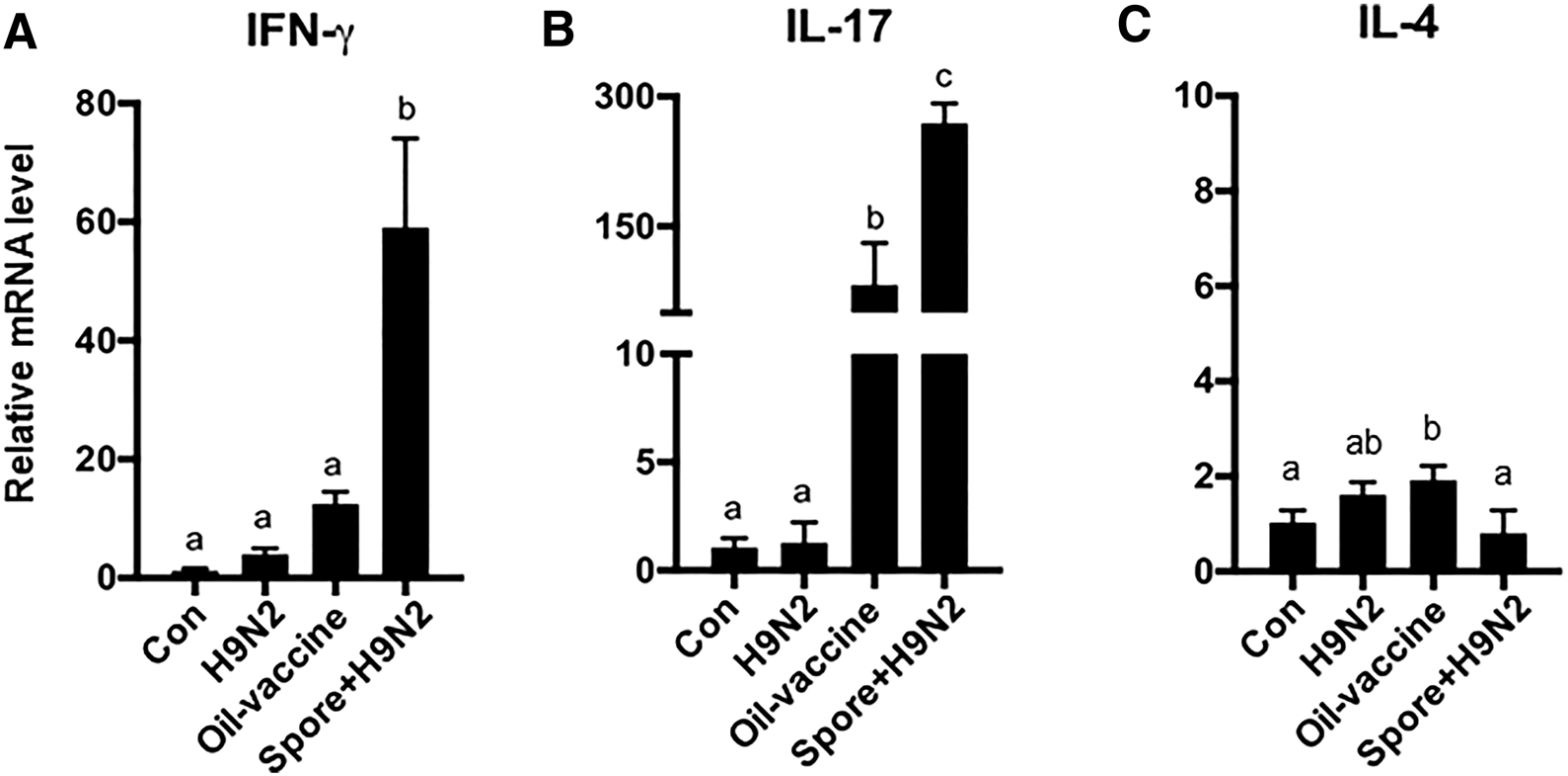
**mRNA expression of cytokines in T cells in spleen from chickens vaccinated with inactivated H9N2 and*B. subtilis*spores.** Chickens (*N* = 5) were administered H9N2 oil vaccine (commercially available), inactivated H9N2 or inactivated H9N2 and *B. subtilis* spores twice at one-week interval. Seven days after the last administration, splenocytes were re-stimulated with inactivated H9N2 for 24 h; then, T cells were isolated by magnetic bead sorting, and mRNA expression of **A** IFN-γ, **B** IL-17 and **C** IL-4 was examined by qRT-PCR. To determine the significance, one-way ANOVA followed by a Friedman test corrected by Dunn’s multiple comparison test was performed. Data are expressed as the mean values ± SDs. Different letters on each group denote a significant difference at *P* ≤ 0.05.

Next, we investigated antigen-specific T cell responses in chickens immunized with inactivated H9N2 with/without *B. subtilis* spores in vivo compared to those in oil vaccine-administered chickens. To investigate H9N2 virus antigen-specific T cell activities, the expression levels of the IFN-γ, IL-4, and IL-17 genes were analysed in CD3^+^ T cells isolated from immunized chickens after re-stimulation with H9N2. The expression levels of IFN-γ (Figure 6A) and IL-17 (Figure 6B) were strongly induced in T cells from chickens immunized with Spore + H9N2 treatment, while minor changes were found in other groups (Figure 6B). The expression levels of IL-4 were relatively low in all groups (Figure 6C). These results demonstrated that *B. subtilis* spores as adjuvants could promote stronger antigen-specific Th1- and Th17-driven immune responses against H9N2 than a commercial oil vaccine.

## Discussion

Adjuvants are now considered as an essential component of most, if not all, inactivated virus vaccines in the poultry industry [29]. The elucidation of the action mechanism of adjuvants has contributed greatly to the development of the appropriate adjuvants in a rational approach to modern vaccine formulation [30, 31]. Although adjuvants have diverse activities [32], the exact role of each adjuvant has not yet been well clarified, especially in chickens. Oil adjuvant, one of the most commonly used adjuvants in domestic animal vaccines, is known to have safety and robust efficacy profiles, with antigen-specific antibodies induced against influenza viruses. It has also been applied to vaccines for low-pathogenicity avian influenza H9N2 vaccines for decades [33, 34]. However, H9N2 has not been conquered worldwide, and moreover, outbreaks have been reported in animals vaccinated against H9N2 infection. It has also been reported that oil adjuvants are not appropriate for inducing T cell responses, which are essential for memory T or B cell generation [35]; therefore, novel adjuvants and formulations of vaccine adjuvants are essential. In the present study, we demonstrated that *B. subtilis* spores could act as potential vaccine adjuvants that synergistically provide antigen-specific immune responses against the avian influenza virus H9N2 in White Leghorn chickens.

Our results showed that *B. subtilis* spores could induce the expression of the pro-inflammatory cytokines IL-1β and IL-6 in innate immune cells such as monocytes and macrophages. These cytokines play a critical role not only in controlling and eliminating invading pathogens but also in provoking the activation of adaptive immune cells such as T cells, especially Th1 and Th17 differentiation [36, 37]. It has been suggested that high expression of pro-inflammatory cytokines upon *B. subtilis* spore treatment is associated with improved resistance to viral infection [12]. We showed that soluble factors such as cytokines from *B. subtilis* spore-treated innate immune cells can enhance the proliferation of T cells, suggesting the importance of cytokines from innate cells treated with spores that subsequently enhance antigen-specific adaptive immune responses. In the present study, upregulation of gene expression of B cell-related genes such as BAFF or CD40L in total splenocytes after *B. subtilis* spore treatment showed a positive correlation with increased B cell numbers. We also showed that the proliferation of CD8^+^ and CD4^+^ T cells was increased by *B. subtilis* spores, which deserves further research to validate and identify the cross-presentation of exogenous antigens in antigen-presenting cells followed by activation of antigen-specific CD8^+^ T cells. On the other hand, since there is no suitable model to examine antigen-specific immune responses in chickens, we need more sophisticated in vitro and in vivo models to evaluate antigen-specific B and T cell responses. Furthermore, antigen-specific T or B cell responses should also be analysed at the protein level or single-cell level using ELISA or intracellular staining and flow cytometry in subsequent studies.

One of the most important factors for vaccine development is probably whether the vaccine induces a long-term immunological memory response. In particular, the induction of proper memory responses should be more important in laying hens and great grandparent stocks that generally stay alive for a longer period of time. The generation of memory responses is largely dependent on the activation of T cells, especially CD4^+^ helper T cells. Most vaccine studies performed in chickens have focused only on humoral responses, including antigen-specific or neutralizing antibody responses; however, the evaluation of T cell quality has been less studied. Our results showed that the commercial oil adjuvant vaccine could not induce a strong T cell response compared to that induced by H9N2 with the *B. subtilis* spore adjuvant. This result is in line with a previous study that demonstrating the ability of *B. subtilis* spores to increase the level of T cell responses [35]. As *B. subtilis* spores induced stronger recall responses than a commercial oil adjuvant-based vaccine, they may have advantages as adjuvants in terms of the generation of antigen-specific memory responses, which should be further investigated in chickens in the industrial field.

As we have demonstrated in the current study, *B. subtilis* spores instruct Th1 and Th17 immune responses rather than Th2 responses when re-stimulated with H9N2, suggesting that antigen-specific T cell responses could be optimal for anti-viral or anti-bacterial responses. It is important to note that the Spore + H9N2 group had much higher levels of Th1 and Th17 responses than the commercial oil vaccine group. Th1 immune responses are considered to be essentially required against viral infection. IL-17 is known to trigger autoimmune disease or anti-bacterial immune responses [38] but is also reported to have a role in anti-viral immune responses [39]. In the same context as our results, IL-6 and IL-1β produced from innate immune cells, such as macrophages, have been reported to be required for IL-17 production. In addition, some studies have suggested that IL-1β can induce both Th1 and Th17 responses [40, 41]. We focused on systemic immune responses, but tissue-specific immune responses have also been considered a primary target for protection against influenza infection. According to mouse studies [13], spore adjuvants could induce lung-specific immune responses when delivered as intranasal vaccines. Therefore, further studies with *B. subtilis* spore adjuvants should be performed not only as a mucosal adjuvant but also with challenge studies against highly pathogenic influenza while comparing with commercially available vaccine adjuvants.

Taken together, the current study results demonstrated that a *B. subtilis* spore adjuvant can effectively induce antigen-specific antibody and IFN-γ- and IL-17-producing T cell immune responses more potently than a traditional oil adjuvant. Therefore, *B. subtilis* spores could be novel and potential vaccine adjuvants against H9N2 avian influenza virus in chickens.

## Data Availability

The data sets used and analysed during the current study are available from the corresponding author upon reasonable request.

## Abbreviations

*B. subtilis*: *Bacillus subtilis*
BAFF: B-cell activating factor
CD: Cluster of differentiation
CFU: Colony-forming unit
Con: Control
CTV: Cell Trace Violet
DNA: Deoxyribonucleic acid
ELISA: Enzyme-linked immunosorbent assay
HI: Haemagglutination inhibition
HRP: Horseradish peroxidase
IgG: Immunoglobulin G
IL: Interleukin
M-MLV: Moloney-murine leukaemia virus
mRNA: Messenger ribonucleic acid
PCR: Polymerase chain reaction
TACI: Transmembrane activator and calcium-modulating cytophilin ligand interactor
TCR: T cell receptor
Th: Helper T cell
TMB: 3,3′,5,5′-Tetramethylbenzidine
TSB: Trypticase soy broth
YE: Yeast extract
